# The 100 Most Cited Papers in Radiotherapy or Chemoradiotherapy for Cervical Cancer: 1990–2020

**DOI:** 10.3389/fonc.2021.642018

**Published:** 2021-09-01

**Authors:** Weiping Wang, Xiaoliang Liu, Dunhuang Wang, Kang Ren, Yuncan Zhou, Ziqi Zhou, Jie Qiu, Fuquan Zhang, Ke Hu

**Affiliations:** Department of Radiation Oncology, Peking Union Medical College Hospital, Chinese Academy of Medical Sciences & Peking Union Medical College, Beijing, China

**Keywords:** cervical cancer, radiation therapy, bibliometric analysis (BA), concurrent chemoradiotherapy, image-guided adaptive brachytherapy

## Abstract

**Objective:**

This work aims to analyze the 100 most cited papers in radiotherapy or chemoradiotherapy for cervical cancer.

**Methods:**

The 100 most cited papers in radiotherapy or chemoradiotherapy between 1990 and 2020 were identified with Thompson Reuters Web of Science citation indexing on August 24, 2020. The publication years, source titles, countries/regions, total citations, and average citations per year were extracted from the Web of Science. The research type and research domain were classified by the authors.

**Results:**

These 100 papers were cited a total of 28,714 times, and the median number of citations was 169.5 (ranging from 116 to 1,700 times). The most cited paper was “Concurrent cisplatin-based radiotherapy and chemotherapy for locally advanced cervical cancer” by Rose et al., with a total citation of 1,700 times. The International Journal of Radiation Oncology Biology Physics (40 papers), Radiotherapy and Oncology (14 papers), and the Journal of Clinical Oncology (12 papers) published the largest number of papers. USA (47 papers), Austria (18 papers), Canada (13 papers), and England (13 papers) contributed the largest number of papers. Image-guided adaptive brachytherapy (IGABT) had the largest number of papers (25 papers). Concurrent chemotherapy was the most cited research domain, with 10,663 total citations and 592.4 citations per paper.

**Conclusion:**

In the present study, we conducted a bibliometric analysis of the 100 most cited papers in radiotherapy or concurrent chemoradiotherapy for cervical cancer in the past 30 years. IGABT and concurrent chemotherapy were the most cited research domains.

## 1 Introduction

Cervical cancer is one of the most common cancers globally, especially in countries with a middle/low human development index (1). Radiotherapy or chemoradiotherapy plays an important role in the treatment of cervical cancer. Adjuvant radiotherapy/concurrent chemoradiotherapy (CCRT) is performed in early-stage cervical cancer patients with risk factors. Definitive CCRT is the standard treatment approach in patients with locally advanced cervical cancer (LACC). Palliative/salvage radiotherapy is conducted in patients with stage IVB or recurrent disease (2). The staging and treatment strategy have changed dramatically in the past three decades. The FIGO staging system was revised in 1994, 2009 (3), 2014 (4), and 2018 (5). Nodal status is incorporated into stage IIIC disease in the last FIGO staging system (5). The treatment strategy for LACC changed from single radiotherapy to concurrent chemoradiotherapy. Brachytherapy was transformed from two-dimensional brachytherapy to MRI-based image-guided adaptive brachytherapy (IGABT). External beam radiation therapy has changed from conventional radiotherapy to intensity-modulated radiation therapy (IMRT) or conformal radiation therapy.

It is a challenging task for researchers to identify the most influential work in a specific area. Bibliometric analysis, also known as citation analysis, is one way to identify the most influential papers. The citation frequency of a paper can reflect its impact. In the present study, we performed a bibliometric analysis of the 100 most cited papers in radiotherapy or chemoradiotherapy for cervical cancer to identify the most influential work in this area.

## 2 Methods

The Thompson Reuters Web of Science citation indexing database was used to identify the most cited papers. The bibliometric analysis was performed on August 24, 2020. The database selected was the Web of Science Core Collection. The time span was 1990–2020. No restriction was placed on the language or document types. The search strategy was as follows: Title = (Cervical NEAR/3 Cancer OR Cervical NEAR/3 Neoplasm* OR Cervical NEAR/3 Carcinoma* OR cervix NEAR/3 cancer OR Cervix NEAR/3 Neoplasm* OR cervix NEAR/3 carcinoma*) AND Title = (brachytherap* OR chemoradi* OR chemo-radiotherap* OR dosimetric OR EBRT OR DVH OR fractionat* OR IGABT OR IGART OR IGRT OR IMRT OR irradiation OR multileaf OR proton-beam OR proton-therapy OR radiation OR radiosurgery OR radiother* OR SBRT OR Tomotherapy OR VMAT). The search results were ranked by the number of times the papers were cited to identify the 100 most cited papers.

The publication years, source titles, and countries/regions of the 100 most cited papers were collected with “Analyze Results” from the Web of Science. The total citations and average citations per year were extracted with “Create Citation Report” from the Web of Science.

Research types and research domains were classified independently by two authors, WW and DW. Discrepancies were resolved by the third author XL. Research types were classified into research articles, guidelines (including recommendations and consensus), reviews, meta-analyses, editorials, and letters. Research domains were classified into brachytherapy (including IGABT and non-IGABT), external beam radiation therapy (including IMRT and extended-field irradiation), chemotherapy (including concurrent chemotherapy and neoadjuvant/adjuvant chemotherapy), radiosensitivity and biomarkers associated with the efficacy of radiation therapy, prognostic factors of survival after radiation therapy, adjuvant radiotherapy *versus* observations after hysterectomy, definitive radiotherapy *versus* surgery, and others.

## 3 Results

There were 6,218 papers that fulfilled the criterion described above. The 100 most cited papers were identified, and they are listed in **Supplementary Table S1**. These 100 papers were cited a total of 28,714 times, and the median number of citations was 169.5 times (range: 116–1,700 times). “Concurrent cisplatin-based radiotherapy and chemotherapy for locally advanced cervical cancer” by Rose et al. (6) had both the highest number of total citations (1,700 times) and the highest number of citations per year (77.27 times). Among the 10 most cited papers (**Table 1**), there were five randomized clinical trials (6–10) and one meta-analysis (11) comparing cisplatin-based concurrent chemoradiotherapy and single radiotherapy in patients with cervical cancer, and there were two recommendations on IGABT released by the gynecological (GYN) GEC ESTRO Working Group (12, 13). The other two papers were randomized clinical trials comparing radical surgery and radiotherapy for stages Ib–IIa cervical cancer (14) and comparing adjuvant radiotherapy and no further therapy after radical hysterectomy and pelvic lymphadenectomy in selected patients with cervical cancer (15).

**Table 1:** The 10 most cited papers in radiotherapy or chemoradiotherapy for cervical cancer from 1990 to 2020.

Rank	Title	Journal	Year	Total citations	Average citations per year (rank)
1	Concurrent cisplatin-based radiotherapy and chemotherapy for locally advanced cervical cancer	N Engl J Med	1999	1,700	77.27 (1)
2	Pelvic radiation with concurrent chemotherapy compared with pelvic and para-aortic radiation for high-risk cervical cancer	N Engl J Med	1999	1,528	69.45 (2)
3	Concurrent chemotherapy and pelvic radiation therapy compared with pelvic radiation therapy alone as adjuvant therapy after radical surgery in high-risk early-stage cancer of the cervix	J Clin Oncol	2000	1,343	63.95 (3)
4	Cisplatin, radiation, and adjuvant hysterectomy compared with radiation and adjuvant hysterectomy for bulky stage IB cervical carcinoma	N Engl J Med	1999	1,337	60.77 (5)
5	Randomized study of radical surgery *versus* radiotherapy for stages Ib–IIa cervical cancer	Lancet	1997	1,054	43.92 (9)
6	Randomized comparison of fluorouracil plus cisplatin *versus* hydroxyurea as an adjunct to radiation therapy in stages IIB–IVA carcinoma of the cervix with negative para-aortic lymph nodes: a Gynecologic Oncology Group and Southwest Oncology Group study	J Clin Oncol	1999	1,028	46.73 (7)
7	Recommendations from Gynaecological (GYN) GEC ESTRO Working Group (II): concepts and terms in 3D image-based treatment planning in cervix cancer brachytherapy—3D dose volume parameters and aspects of 3D image-based anatomy, radiation physics, radiobiology	Radiother Oncol	2006	958	63.87 (4)
8	Recommendations from Gynaecological (GYN) GEC-ESTRO Working Group* (I): concepts and terms in 3D image based 3D treatment planning in cervix cancer brachytherapy with emphasis on MRI assessment of GTV and CTV	Radiother Oncol	2005	882	55.13 (6)
9	Survival and recurrence after concomitant chemotherapy and radiotherapy for cancer of the uterine cervix: a systematic review and meta-analysis	Lancet	2001	750	37.5 (11)
10	A randomized trial of pelvic radiation therapy *versus* no further therapy in selected patients with stage IB carcinoma of the cervix after radical hysterectomy and pelvic lymphadenectomy: a Gynecologic Oncology Group study	Gynecol Oncol	1999	642	29.18 (14)

### 3.1 Time Distribution and Article Type

**Figure 1:**
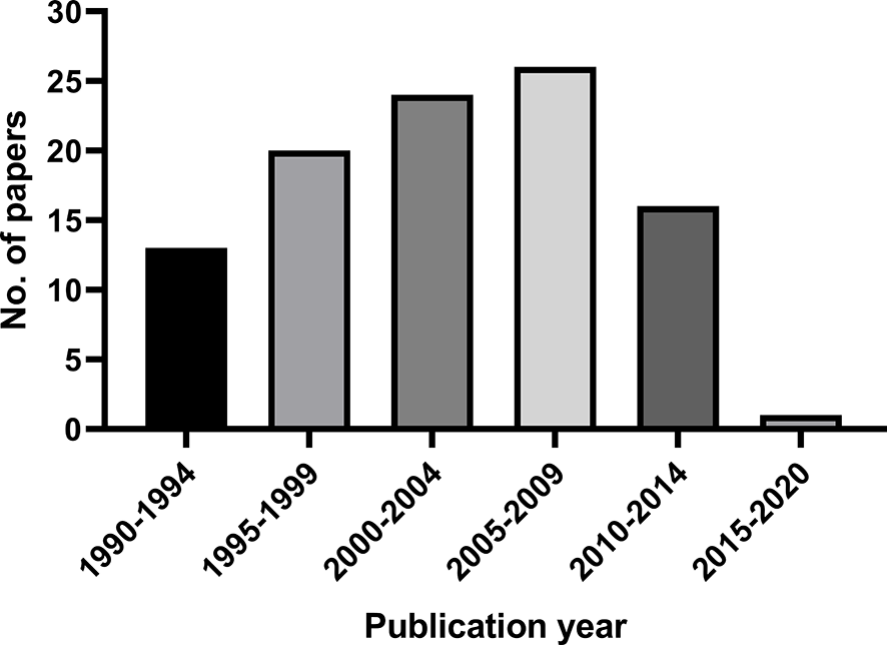
The publication time distribution of the 100 most cited papers in radiotherapy or concurrent chemoradiotherapy for cervical cancer between 1990 and 2020.

These most cited 100 papers were published between 1990 and 2016. The publication time distribution is shown in **Figure 1**. The largest number of papers were published during 2005–2009 (26 papers), followed by 2000–2004 (24 papers) and 1995–1999 (20 papers).

Among these papers, there were 83 research articles, 10 guidelines, recommendations, or consensuses, six meta-analyses, and one editorial. Of the 83 research articles, 20 were randomized clinical trials (RCTs). The average citations per paper of RCTs, meta-analysis, and non-RCT research articles were 584.8, 357.7, and 180.5, respectively.

### 3.2 Journals

**Figure 2:**
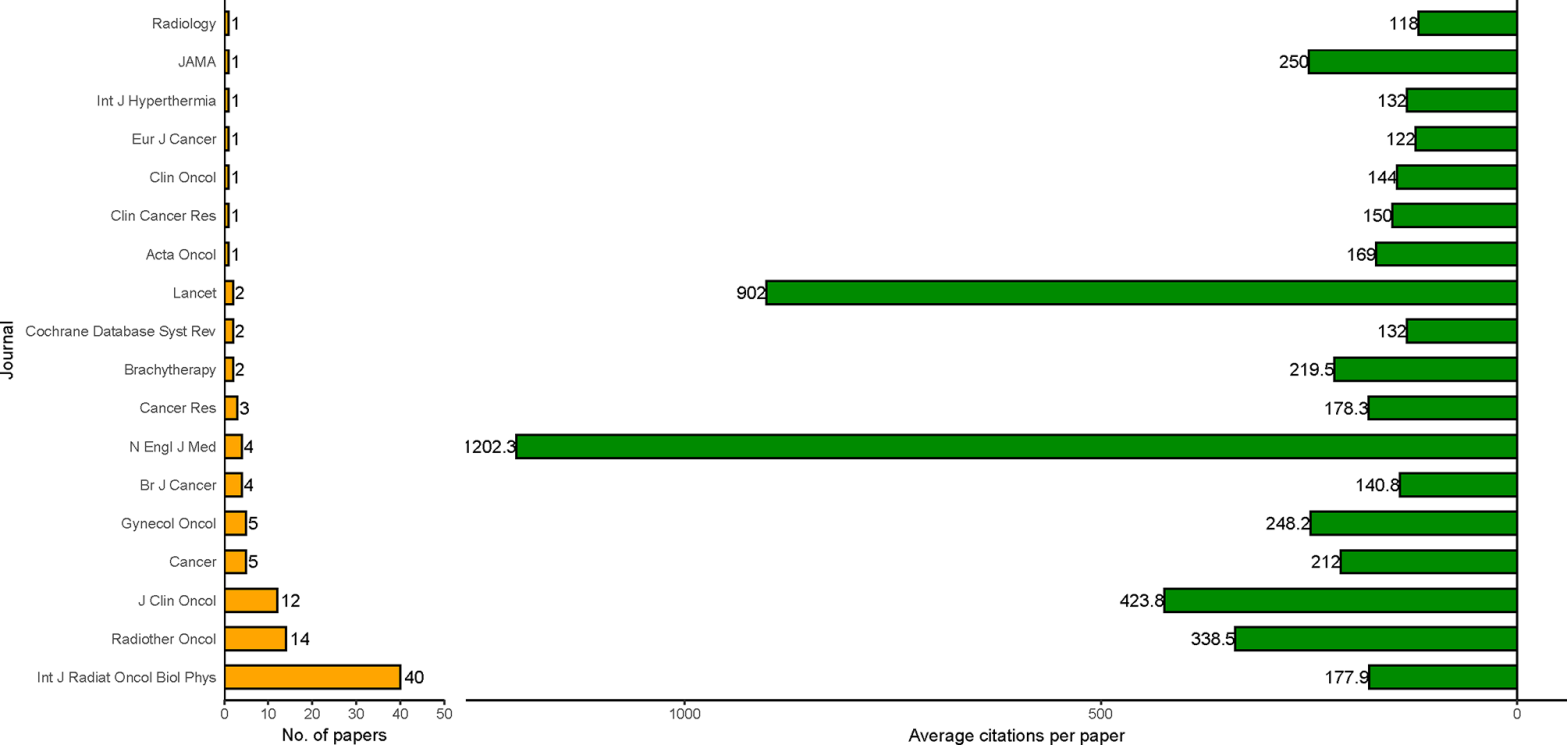
Journals of the 100 most cited papers in radiotherapy or concurrent chemoradiotherapy for cervical cancer between 1990 and 2020. Average citation paper = total citations/number of papers.

As shown in **Figure 2**, the International Journal of Radiation Oncology Biology Physics published the largest number of papers (40 papers), followed by Radiotherapy and Oncology (14 papers) and the Journal of Clinical Oncology (12 papers). New England Journal of Medicine (1,202.3 citations per paper) and Lancet (902 citations per paper) had the largest average citations per paper.

### 3.3 Countries

**Figure 3:**
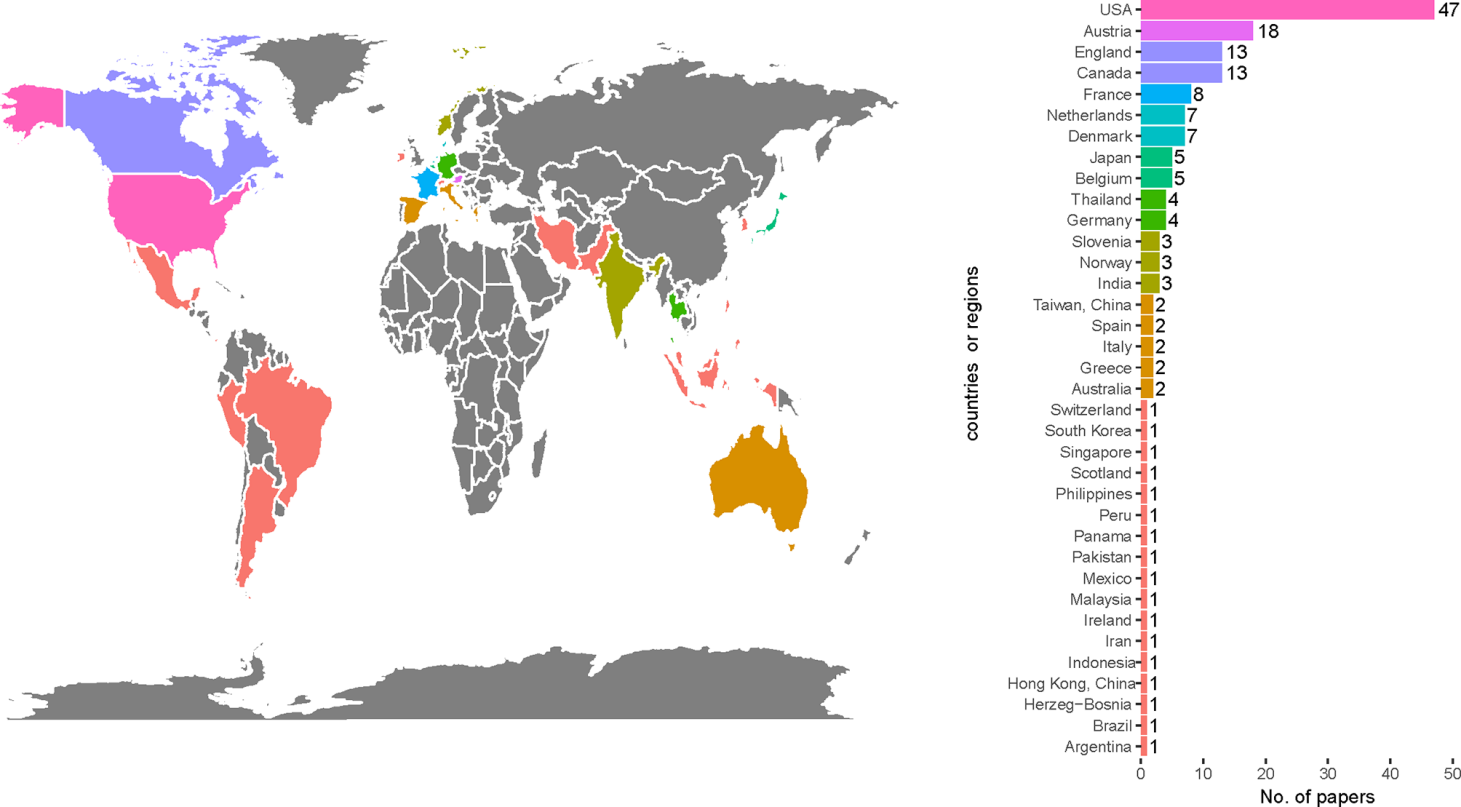
Most frequent countries or regions participating in the 100 most cited papers in radiotherapy or concurrent chemoradiotherapy in cervical cancer between 1990 and 2020.

A total of 36 countries or regions participated in the publication of the 100 most cited papers. Most of them are developed countries. As shown in **Figure 3**, USA contributed the most papers (47 papers), followed by Austria (18 papers), Canada (13 papers), and England (13 papers).

### 3.4 Research Domains

The research domains of these papers are shown in **Table 2**. In the area of brachytherapy, chemotherapy, and external beam radiation therapy, there were 32 papers (7,594 citations), 21 papers (10,873 citations), and 13 papers (4,113 papers), respectively.

**Table 2:** Research domains of the 100 most cited papers in radiotherapy or concurrent chemoradiotherapy for cervical cancer between 1990 and 2020.

Research domains	Number of articles	Total number of citations	Average citations per year (per paper)	Publication year
Brachytherapy	32	7,594	237.3	1991–2016
Image-guided adaptive brachytherapy (IGABT)	25	6,383	255.3	2003–2016
Brachytherapy (non-IGABT)	8	1,436	179.5	1991–2014
Chemotherapy	21	10,873	517.8	1991–2011
Concurrent chemoradiotherapy *versus* radiotherapy	18	10,663	592.4	1998–2010
Neoadjuvant/adjuvant chemotherapy	5	1,323	264.6	1991–2011
External beam radiation therapy	13	4,113	316.4	1995–2011
Intensity-modulated radiation therapy	8	1,424	178.0	2001–2011
Extended field radiation therapy	5	2,689	537.8	1995–2008
Radiosensitivity and biomarkers associated with efficacy of radiation therapy	11	2,030	184.5	1991–2004
Prognostic factors of survival after radiation therapy	11	2,350	213.6	1991–2008
Adjuvant radiotherapy *versus* observation after hysterectomy	3	1,042	347.3	1990–2006
Definitive radiotherapy *versus* surgery	3	1,490	49.7	1997–2003
Others	11	1,587	144.3	1992–2009

Some papers belonged to two or more domains.

In the subdivided research domains, IGABT had the largest number of papers (25 papers). Concurrent chemotherapy was the most cited research domain, with 10,663 total citations and 592.4 citations per paper.

**Figure 4:**
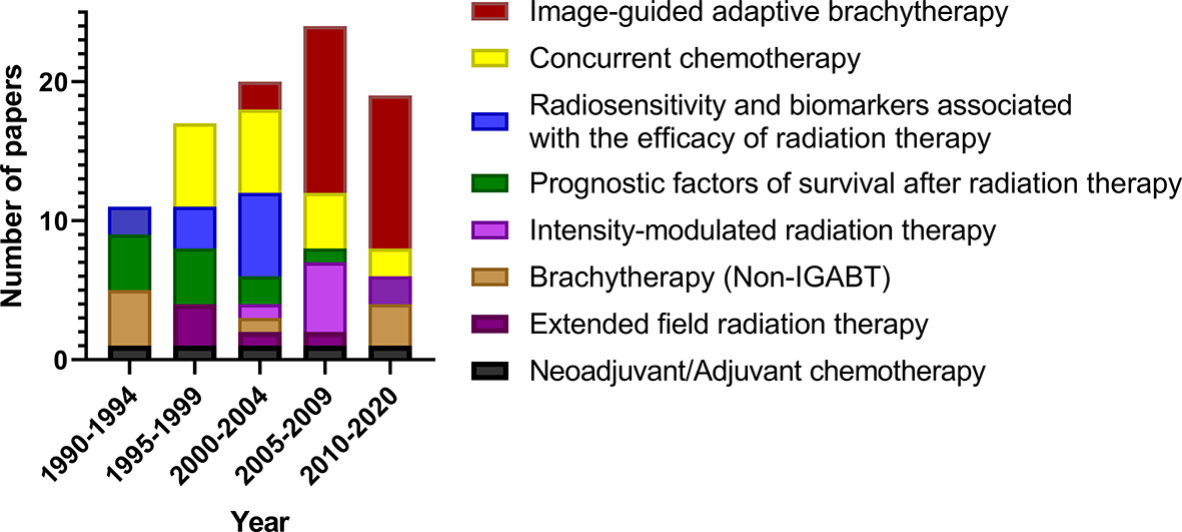
Main research domains in different time periods of the 100 most cited papers in radiotherapy or concurrent chemoradiotherapy for cervical cancer between 1990 and 2020.

The main research domain in different time periods is shown in **Figure 4**. From 1990 to 2020, studies on the prognostic factors of survival after radiotherapy, radiosensitivity, and biomarkers associated with the efficacy of radiation therapy decreased. There were more studies on IGABT and IMRT.

#### 3.4.1 Concurrent Chemotherapy

All of the 18 papers on concurrent chemotherapy were published between 1998 and 2010. Of them, there were 11 RCTs, six meta-analyses, and one editorial. Eight of them were published in the Journal of Clinical Oncology, followed by the New England Journal of Medicine (four papers) and the Cochrane Database of Systematic Reviews (two papers). The USA participated in 10 of these papers, followed by Canada (five papers), England (four papers), and Thailand (two papers).

#### 3.4.2 IGABT

All 25 papers on IGABT were published between 2003 and 2016. Of them, 11 papers were published in the International Journal of Radiation Oncology Biology Physics, 11 papers were published in Radiation and Oncology, two papers were published in Brachytherapy, and one paper was published in Acta Oncology. Austria participated in 17 of these papers, which was the most, followed by France (seven papers), Denmark (six papers), the USA (six papers), and Belgium (five papers).

## 4 Discussion

Of the 100 most cited papers in radiotherapy for cervical cancer, 18 papers focused on concurrent chemotherapy. The total number of citations of these 18 papers on CCRT *versus* single radiotherapy was 10,663, which was the largest. Before 1999, single radiotherapy or radiotherapy combined with hydroxyurea was performed for cervical cancer. Between 1999 and 2000, five randomized clinical trials demonstrated that cisplatin-based CCRT was superior to radiotherapy (6–10) (ranked first, cited 1,700 times; ranked second, cited 1,528 times; ranked third, cited 1,343 times; ranked 4th, cited 1,337 times; ranked 6th, cited 1,028 times). In 2001, a meta-analysis demonstrated that CCRT improved the overall survival (OS) and progression-free survival (PFS) of patients with cervical cancer. The absolute benefit of OS and PFS were 12 and 16%, respectively (11) (ranked 9th, cited 750 times). These studies (6–11) established the role of CCRT in patients with cervical cancer. Twenty years have passed and CCRT is still the standard treatment in patients with locally advanced cervical cancer treated with definitive radiotherapy and for patients with high-risk factors after radical hysterectomy (2). All these 18 papers on CCRT were published before 2010. In the past decade, the role of CCRT has been established and the number of influential studies decreased.

In the domain of concurrent chemotherapy for cervical cancer, journals in the area of oncology or general medicine, the Journal of Clinical Oncology and the New England Journal of Medicine, published the largest number of papers. This is different than the most frequent journals among these 100 papers, the International Journal of Radiation Oncology Biology Physics and Radiation Oncology, two journals focused on radiotherapy. The USA made a great contribution to this area. Institutes or authors from the USA participated in nine of the 17 most cited papers.

In the past several decades, brachytherapy for cervical cancer changed from two-dimensional brachytherapy to MRI or CT-based IGABT. Thirty-two of the 100 most cited papers focused on brachytherapy, which was the largest number. Of them, 25 papers focused on IGABT. The Medical University of Vienna, an institute located in Austria, made great contributions to this area. In the most cited papers in this domain, there were seven recommendations or consensus guidelines: four from the gynecological (GYN) GEC ESTRO Working Group (12, 13, 16, 17) (ranked 7th, cited 958 times; ranked 8th, cited 882 times; ranked 35th, cited 214 times; ranked 53rd, cited 169 times), two from the American Brachytherapy Society (ranked 32nd, cited 225 times; ranked 36th, cited 214 times) (18, 19), and one from the Image-Guided Brachytherapy Working Group (20) (ranked 57th, cited 160 times). These recommendations or guidelines contributed to the progress and standardization of IGABT. RetroEMBRACE, EMBRACE, and the new EMBRACE II were the most impactful studies in the domain of IGABT. The results of retroEMBRACE (21) were reported in 2016 in the 40th most cited paper, with an average of 41.8 citations per year (ranked 10th). As most RetroEMBRACE-, EMBRACE-, and EMBRACE II-based studies were reported after 2015, some of them may become the most cited papers 5 or 10 years later. The International Journal of Radiation Oncology Biology Physics and Radiotherapy and Oncology were the most impactful journals for IGABT, publishing 22 of the 25 papers. More impactful papers on brachytherapy in cervical cancer were reported by Tang et al., with the 100 most cited papers in brachytherapy for cervical cancer (22).

IMRT is an important technical advancement in radiotherapy. Compared with conventional or conformal radiotherapy, for cervical cancer, IMRT is superior in sparing the bowel, rectum, and bladder (23, 24) (ranked 18th, cited 322 times; ranked 67th, cited 143 times) and bone marrow (25) (ranked 97th, cited 117 times). IMRT is recommended as adjuvant radiotherapy in patients with cervical cancer (2, 26). The Radiation Therapy Oncology Group led an international collaboration of cooperative groups and released a consensus guideline for target volume delineation for IMRT in postoperative radiotherapy in patients with cervical or endometrial cancer (27) (ranked 37th, cited 213 times). However, considering organ motion (28) (ranked 78th, cited 129 times) and tumor regression, IMRT has not been recommended as definitive radiotherapy for patients with cervical cancer for a long time. In 2010, Kidd et al. reported that PET/CT-guided IMRT had improved the survival and was less toxic than non-IMRT radiotherapy in patients with LACC treated with definitive radiotherapy (29) (ranked 96th, cited 117 time). In 2011, the Gyn IMRT Consortium released consensus guidelines for target volume delineation for IMRT in patients with cervical cancer treated with definitive radiotherapy (30) (ranked 38th, cited 212 times). In recent years, additional studies have demonstrated that IMRT is associated with less toxicity and comparable clinical outcomes compared with conventional or conformal radiotherapy in patients with cervical cancer treated with definitive radiotherapy (31–35). In the 2020 ASTRO clinical practice guideline, IMRT is conditionally recommended as definitive radiotherapy for cervical cancer (26).

We identified hotspots in the area of radiotherapy or chemoradiotherapy in cervical cancer using the 100 most cited papers. CCRT and adjuvant radiotherapy improved the survival of patients with cervical cancer. IGABT and IMRT reduce the toxicity of radiotherapy. The local control of LACC is excellent with CCRT, IGABT, and IMRT (21, 35). At present, there are more distant failures than local failures (35). Adjuvant chemotherapy, neo-adjuvant chemotherapy, and prophylactic para-aortic lymph node irradiation are potential ways to reduce distant metastasis. Recently, the OUTBACK trial demonstrated that adjuvant chemotherapy following chemoradiotherapy did not improve the survival of patients with LACC (36). The results of the neoadjuvant chemotherapy and prophylactic para-aortic lymph node irradiation were obtained from INTERLACE (NCT01566240) and NCT03955367, *etc*. Immunotherapy and targeted therapy are also potential ways to prevent distant metastasis of LACC in the future.

This study has some limitations. First, the citations of papers are influenced by many factors, including types of papers, journals, publication time, influence of authors, and even social media exposure. The citation of papers was influenced by the time span after publication. As a result, eight of the 10 most cited papers were published before 2001, and 83 of the 100 most cited papers were published before 2009. No papers published after 2017 were ranked among the top 100 papers. This may lead to missing impactful studies published in the past few years. The number of citations per year was used in the present study to balance the impact of the time span after publication on the selected papers. Second, some highly cited papers are not applicable or insignificant at present. For example, in 1990, Soisson et al. conducted a nonrandomized study that demonstrated that adjuvant radiotherapy following radical hysterectomy had limited benefit for patients with stage IB and IIA cervical cancer compared with radical hysterectomy alone (37) (ranked 77th, cited 130 times). However, adjuvant radiotherapy has been established as the standard treatment after radical hysterectomy for cervical cancer patients meeting the Sedlis criteria. The conclusion of the study of Soisson et al. study may not be applicable at present. Third, only the Web of Science citation indexing database was used to identify the most cited papers. With only 100 papers from the Web of Science, we may miss some influential papers. Fourth, we tried our best to summarize the research domains. However, in the bibliometric analysis, it is difficult to analyze the detailed contents and study design of the papers.

## Conclusion

In the present study, we conducted a bibliometric analysis of the 100 most cited papers in radiotherapy or concurrent chemoradiotherapy for cervical cancer in the past 30 years. IGABT and concurrent chemotherapy were the most cited research domains.

## Data Availability Statement

The original contributions presented in the study are included in the article/**Supplementary Material**. Further inquiries can be directed to the corresponding authors.

## Author Contributions

FZ and KH contributed to the conception of the study. WW and XL wrote the first draft of the manuscript. DW, KR, YZ, ZZ and JQ contributed to the review of literatures. All authors contributed to the article and approved the submitted version.

## Funding

This work was supported by the National Natural Science Foundation of China (grant number U19A2064) and the National Key Technologies Research and Development Program of China (grant number 2016YFC0105207).

## Conflict of Interest

The authors declare that the research was conducted in the absence of any commercial or financial relationships that could be construed as a potential conflict of interest.

## Publisher’s Note

All claims expressed in this article are solely those of the authors and do not necessarily represent those of their affiliated organizations, or those of the publisher, the editors and the reviewers. Any product that may be evaluated in this article, or claim that may be made by its manufacturer, is not guaranteed or endorsed by the publisher.
