# Supplementary material for: The 100 Most Cited Papers in Radiotherapy or Chemoradiotherapy for Cervical Cancer: 1990–2020
Source: Front Oncol. 2021 Sep 1;11:642018. doi: 10.3389/fonc.2021.642018 (PMC8440989; doi:10.3389/fonc.2021.642018)
Supplement: Supplementary file 1 [file Table_1.docx]

Supplementary Table 1. The 100 most cited papers in radiotherapy or chemoradiotherapy for cervical cancer from 1990 to 2020

| Rank | Title | Journal | Year | Total citation | Average citation per year (rank) |
| --- | --- | --- | --- | --- | --- |
| 1 | Concurrent cisplatin-based radiotherapy and chemotherapy for locally advanced cervical cancer | N Engl J Med | 1999 | 1700 | 77.27(1) |
| 2 | Pelvic radiation with concurrent chemotherapy compared with pelvic and para-aortic radiation for high-risk cervical cancer | N Engl J Med | 1999 | 1528 | 69.45(2) |
| 3 | Concurrent chemotherapy and pelvic radiation therapy compared with pelvic radiation therapy alone as adjuvant therapy after radical surgery in high-risk early-stage cancer of the cervix | J Clin Oncol | 2000 | 1343 | 63.95(3) |
| 4 | Cisplatin, radiation, and adjuvant hysterectomy compared with radiation and adjuvant hysterectomy for bulky stage IB cervical carcinoma | N Engl J Med | 1999 | 1337 | 60.77(5) |
| 5 | Randomised study of radical surgery versus radiotherapy for stage Ib-IIa cervical cancer | Lancet | 1997 | 1054 | 43.92(9) |
| 6 | Randomized comparison of fluorouracil plus cisplatin versus hydroxyurea as an adjunct to radiation therapy in stage IIB-IVA carcinoma of the cervix with negative para-aortic lymph nodes: A Gynecologic Oncology Group and Southwest Oncology Group Study | J Clin Oncol | 1999 | 1028 | 46.73(7) |
| 7 | Recommendations from gynaecological (GYN) GEC ESTRO working group (II): Concepts and terms in 3D image-based treatment planning in cervix cancer brachytherapy - 3D dose volume parameters and aspects of 3D image-based anatomy, radiation physics, radiobiotogy | Radiother Oncol | 2006 | 958 | 63.87(4) |
| 8 | Recommendations from Gynaecological (GYN) GEC-ESTRO Working Group* (I): concepts and terms in 3D image based 3D treatment planning in cervix cancer brachytherapy with emphasis on MRI assessment of GTV and CTV | Radiother Oncol | 2005 | 882 | 55.13(6) |
| 9 | Survival and recurrence after concomitant chemotherapy and radiotherapy for cancer of the uterine cervix: a systematic review and meta-analysis | Lancet | 2001 | 750 | 37.5(11) |
| 10 | A randomized trial of pelvic radiation therapy versus no further therapy in selected patients with stage is carcinoma of the cervix after radical hysterectomy and pelvic lymphadenectomy: A gynecologic oncology group study | Gynecol Oncol | 1999 | 642 | 29.18(14) |
| 11 | Pelvic irradiation with concurrent chemotherapy versus pelvic and para-aortic irradiation for high-risk cervical cancer: An update of radiation therapy oncology group trial (RTOG) 90-01 | J Clin Oncol | 2004 | 632 | 37.18(12) |
| 12 | Oxygenation predicts radiation response and survival in patients with cervix cancer | Radiother Oncol | 1998 | 513 | 22.3(22) |
| 13 | Reducing Uncertainties About the Effects of Chemoradiotherapy for Cervical Cancer: A Systematic Review and Meta-Analysis of Individual Patient Data From 18 Randomized Trials | J Clin Oncol | 2008 | 471 | 36.23(13) |
| 14 | Clinical outcome of protocol based image (MRI) guided adaptive brachytherapy combined with 3D conformal radiotherapy with or without chemotherapy in patients with locally advanced cervical cancer | Radiother Oncol | 2011 | 453 | 45.3(8) |
| 15 | Clinical impact of MRI assisted dose volume adaptation and dose escalation in brachytherapy of locally advanced cervix cancer | Radiother Oncol | 2007 | 361 | 25.79(16) |
| 16 | Phase III trial comparing radical radiotherapy with and without cisplatin chemotherapy in patients with advanced squamous cell cancer of the cervix | J Clin Oncol | 2002 | 331 | 17.42(28) |
| 17 | The American Brachytherapy Society recommendations for high-dose-rate brachytherapy for carcinoma of the cervix | Int J Radiat Oncol Biol Phys | 2000 | 330 | 15.71(31) |
| 18 | Intensity-modulated radiation therapy (IMRT) reduces small bowel, rectum, and bladder doses in patients with cervical cancer receiving pelvic and para-aortic irradiation | Int J Radiat Oncol Biol Phys | 2001 | 322 | 16.1(30) |
| 19 | Carcinoma of the cervix treated with radiation therapy-I - a multi-variate analysis of prognostic variables in the gynecologic oncology group | Cancer | 1991 | 318 | 10.6(53) |
| 20 | Computed tomography versus magnetic resonance imaging-based contouring in cervical cancer brachytherapy: Results of a prospective trial and preliminary guidelines for standardized contours | Int J Radiat Oncol Biol Phys | 2007 | 314 | 22.43(21) |
| 21 | The importance of hemoglobin levels during radiotherapy for carcinoma of the cervix | Cancer | 1999 | 283 | 12.86(42) |
| 22 | A phase III randomized trial of postoperative pelvic irradiation in stage ib cervical carcinoma with poor prognostic features: Follow-up of a gynecologic oncology group study | Int J Radiat Oncol Biol Phys | 2006 | 270 | 18(27) |
| 23 | Radiation sensitivity, H2AX phosphorylation, and kinetics of repair of DNA strand breaks in irradiated cervical cancer cell lines | Cancer Research | 2004 | 265 | 15.59(32) |
| 24 | Carcinoma of the uterine cervix .1. impact of prolongation of overall treatment time and timing of brachytherapy on outcome of radiation-therapy | Int J Radiat Oncol Biol Phys | 1995 | 263 | 10.12(56) |
| 25 | Neoadjuvant chemotherapy and radical surgery versus exclusive radiotherapy in locally advanced squamous cell cervical cancer: Results from the Italian Multicenter Randomized study | J Clin Oncol | 2002 | 261 | 13.74(37) |
| 26 | Time-course and incidence of late complications in patients treated with radiation-therapy for FIGO stage Ib carcinoma of the uterine cervix | Int J Radiat Oncol Biol Phys | 1995 | 259 | 9.96(57) |
| 27 | Prophylactic extended-field irradiation of paraaortic lymph-nodes in stage-IIb and bulky stage-Ib and stage-IIa cervical carcinomas - 10-year treatment results of RTOG-79-20 | Jama | 1995 | 250 | 9.62(61) |
| 28 | Phase III, Open-Label, Randomized Study Comparing Concurrent Gemcitabine Plus Cisplatin and Radiation Followed by Adjuvant Gemcitabine and Cisplatin Versus Concurrent Cisplatin and Radiation in Patients With Stage IIB to IVA Carcinoma of the Cervix | J Clin Oncol | 2011 | 248 | 24.8(18) |
| 29 | Dose and volume parameters for MRI-based treatment planning in intracavitary brachytherapy for cervical cancer | Int J Radiat Oncol Biol Phys | 2005 | 245 | 15.31(34) |
| 30 | Improved treatment for cervical, cancer - Concurrent chemotherapy and radiotherapy | N Engl J Med | 1999 | 244 | 11.09(48) |
| 31 | The influence of treatment time on outcome for squamous-cell cancer of the uterine cervix treated with radiation - a patterns-of-care study | Int J Radiat Oncol Biol Phys | 1993 | 239 | 8.54(66) |
| 32 | American Brachytherapy Society consensus guidelines for locally advanced carcinoma of the cervix. Part II: High-dose-rate brachytherapy | Brachytherapy | 2012 | 225 | 25(17) |
| 33 | Measurement of tumor volume by PET to evaluate prognosis in patients with advanced cervical cancer treated by radiation therapy | Int J Radiat Oncol Biol Phys | 2002 | 221 | 11.63(46) |
| 34 | Trends in the Utilization of Brachytherapy in Cervical Cancer in the United States | Int J Radiat Oncol Biol Phys | 2013 | 219 | 27.38(15) |
| 35 | Recommendations from Gynaecological (GYN) GEC-ESTRO Working Group (IV): Basic principles and parameters for MR imaging within the frame of image based adaptive cervix cancer brachytherapy | Radiother Oncol | 2012 | 214 | 23.78(19) |
| 36 | American Brachytherapy Society consensus guidelines for locally advanced carcinoma of the cervix. Part I: General principles | Brachytherapy | 2012 | 214 | 23.78(20) |
| 37 | Consensus guidelines for delineation of clinical target volume for intensity-modulated pelvic radiotherapy in postoperative treatment of endometrial and cervical cancer | Int J Radiat Oncol Biol Phys | 2008 | 213 | 16.38(29) |
| 38 | Consensus guidelines for delineation of clinical target volume for intensity-modulated pelvic radiotherapy for the definitive treatment of cervix cancer | Int J Radiat Oncol Biol Phys | 2011 | 212 | 21.2(24) |
| 39 | Effect of tumor size on the prognosis of carcinoma of the uterine cervix treated with irradiation alone | Cancer | 1992 | 211 | 7.28(76) |
| 40 | Image guided brachytherapy in locally advanced cervical cancer: Improved pelvic control and survival in RetroEMBRACE, a multicenter cohort study | Radiother Oncol | 2016 | 209 | 41.8(10) |
| 41 | A randomized trial of chemotherapy followed by pelvic radiation-therapy in stage-IIIb carcinoma of the cervix | J Clin Oncol | 1991 | 204 | 6.8(79) |
| 42 | A systematic review of acute and late toxicity of concomitant chemoradiation for cervical cancer | Radiother Oncol | 2003 | 197 | 10.94(49) |
| 43 | Impact of 3D image-based PDR brachytherapy on outcome of patients treated for cervix carcinoma in France: Results of the French STIC prospective study | Radiother Oncol | 2012 | 195 | 21.67(23) |
| 44 | Longitudinal study of sexual function and vaginal changes after radiotherapy for cervical cancer | Int J Radiat Oncol Biol Phys | 2003 | 195 | 10.83(51) |
| 45 | The Vienna applicator for combined intracavitary and interstitial brachytherapy of cervical cancer: Design, application, treatment planning, and dosimetric results | Int J Radiat Oncol Biol Phys | 2006 | 192 | 12.8(43) |
| 46 | FIGO IIIB squamous cell carcinoma of the cervix: An analysis of prognostic factors emphasizing the balance between external beam and intracavitary radiation therapy | Int J Radiat Oncol Biol Phys | 1999 | 183 | 8.32(70) |
| 47 | MRI-guided 3D optimization significantly improves DVH parameters of pulsed-dose-rate brachytherapy in locally advanced cervical cancer | Int J Radiat Oncol Biol Phys | 2008 | 177 | 13.62(38) |
| 48 | Dose-effect relationship for local control of cervical cancer by magnetic resonance image-guided brachytherapy | Radiother Oncol | 2009 | 175 | 14.58(35) |
| 49 | Radiation therapy with and without extrafascial hysterectomy for bulky stage IB cervical carcinoma: a randomized trial of the Gynecologic Oncology Group | Gynecol Oncol | 2003 | 175 | 9.72(59) |
| 50 | Dosimetric predictors of acute hematologic toxicity in cervical cancer patients treated with concurrent cisplatin and intensity-modulated pelvic radiotherapy | Int J Radiat Oncol Biol Phys | 2006 | 171 | 11.4(47) |
| 51 | Tumor size, irradiation dose, and long-term outcome of carcinoma of uterine cervix | Int J Radiat Oncol Biol Phys | 1998 | 170 | 7.39(73) |
| 52 | MRI-guided adaptive radiotherapy in locally advanced cervical cancer from a Nordic perspective | Acta Oncol | 2013 | 169 | 21.13(25) |
| 53 | Recommendations from Gynaecological (GYN) GEC-ESTRO Working Group: Considerations and pitfalls in commissioning and applicator reconstruction in 3D image-based treatment planning of cervix cancer brachytherapy | Radiother Oncol | 2010 | 169 | 15.36(33) |
| 54 | Diffusion-weighted magnetic resonance imaging in the early detection of response to chemoradiation in cervical cancer | Gynecol Oncol | 2008 | 169 | 13(41) |
| 55 | Dose-volume histogram parameters and local tumor control in magnetic resonance image-guided cervical cancer brachytherapy | Int J Radiat Oncol Biol Phys | 2009 | 163 | 13.58(39) |
| 56 | Cervical carcinoma metastatic to para-aortic nodes: Extended field radiation therapy with concomitant 5-fluorouracil and cisplatin chemotherapy: A Gynecologic Oncology Group Study | Int J Radiat Oncol Biol Phys | 1998 | 163 | 7.09(77) |
| 57 | Proposed guidelines for image-based intracavitary brachytherapy for cervical carcinoma: Report from Image-Guided Brachytherapy Working Group | Int J Radiat Oncol Biol Phys | 2004 | 160 | 9.41(63) |
| 58 | Hypoxia-induced treatment failure in advanced squamous cell carcinoma of the uterine cervix is primarily due to hypoxia induced radiation resistance rather than hypoxia-induced metastasis | Br J Cancer | 2000 | 155 | 7.38(74) |
| 59 | Long-term follow-up of a randomized trial comparing concurrent single agent cisplatin, cisplatin-based combination chemotherapy, or hydroxyurea during pelvic irradiation for locally advanced cervical cancer: A gynecologic oncology group study | J Clin Oncol | 2007 | 153 | 10.93(50) |
| 60 | The Vienna applicator for combined intracavitary and interstitial brachytherapy of cervical cancer: Clinical feasibility and preliminary results | Int J Radiat Oncol Biol Phys | 2006 | 153 | 10.2(54) |
| 61 | Intrinsic radiosensitivity and prediction of patient response to radiotherapy for carcinoma of the cervix | Br J Cancer | 1993 | 153 | 5.46(88) |
| 62 | Overexpression of hypoxia-inducible factor 1 alpha indicates diminished response to radiotherapy and unfavorable prognosis in patients receiving radical radiotherapy for cervical cancer | Clin Cancer Res | 2003 | 150 | 8.33(68) |
| 63 | Interstitial hypertension in carcinoma of uterine cervix in patients: possible correlation with tumor oxygenation and radiation response | Cancer Res | 1991 | 150 | 5(94) |
| 64 | From point A to the sculpted pear: MR image guidance significantly improves tumour dose and sparing of organs at risk in brachytherapy of cervical cancer | Radiother Oncol | 2010 | 145 | 13.18(40) |
| 65 | Distant metastases after irradiation alone in carcinoma of the uterine cervix | Int J Radiat Oncol Biol Phys | 1992 | 145 | 5(95) |
| 66 | Concurrent cisplatin-based chemotherapy plus radiotherapy for cervical cancer - a meta-analysis | Clin Oncol | 2002 | 144 | 7.58(72) |
| 67 | Conventional, conformal, and intensity-modulated radiation therapy treatment planning of external beam radiotherapy for cervical cancer: The impact of tumor regression | Int J Radiat Oncol Biol Phys | 2006 | 143 | 9.53(62) |
| 68 | Comparison of high and low-dose rate remote afterloading for cervix cancer and the importance of fractionation | Int J Radiat Oncol Biol Phys | 1991 | 143 | 4.77(96) |
| 69 | Apoptosis, intrinsic radiosensitivity and prediction of radiotherapy response in cervical-carcinoma | Radiother Oncol | 1995 | 142 | 5.46(89) |
| 70 | Randomized trial of epirubicin and cisplatin chemotherapy followed by pelvic radiation in locally advanced cervical-cancer | J Clin Oncol | 1995 | 139 | 5.35(91) |
| 71 | Low-dose rate vs high-dose-rate brachytherapy in the treatment of carcinoma of the uterine cervix - a clinical-trial | Int J Radiat Oncol Biol Phys | 1994 | 139 | 5.15(92) |
| 72 | Concomitant chemotherapy and radiation therapy for cancer of the uterine cervix | Cochrane Database Syst Rev | 2005 | 136 | 8.5(67) |
| 73 | Radiation therapy morbidity in carcinoma of the uterine cervix: Dosimetric and clinical correlation | Int J Radiat Oncol Biol Phys | 1999 | 136 | 6.18(85) |
| 74 | A randomized clinical trial of radiation therapy versus thermoradiotherapy in stage IIIB cervical carcinoma | Int J Hyperthermia | 2001 | 132 | 6.6(80) |
| 75 | HIGH-DOSE-RATE REMOTE AFTERLOADING INTRACAVITARY RADIATION-THERAPY FOR CANCER OF THE UTERINE CERVIX - A 20-YEAR EXPERIENCE | Cancer | 1992 | 132 | 4.55(97) |
| 76 | Randomized comparison of weekly cisplatin or protracted venous infusion of fluorouracil in combination with pelvic radiation in advanced cervix cancer: A gynecologic oncology group study | J Clin Oncol | 2005 | 130 | 8.13(71) |
| 77 | Adjuvant radiotherapy following radical hysterectomy for patients with stage Ib and IIa cervical cancer | Gynecol Oncol | 1990 | 130 | 4.19(99) |
| 78 | Inter- and intrafractional tumor and organ movement in patients with cervical cancer undergoing radiotherapy: A cinematic-MRI point-of-interest study | Int J Radiat Oncol Biol Phys | 2008 | 129 | 9.92(58) |
| 79 | The independence of intrinsic radiosensitivity as a prognostic factor for patient response to radiotherapy of carcinoma of the cervix | Br J Cancer | 1997 | 129 | 5.38(90) |
| 80 | Reducing uncertainties about the effects of chemoradiotherapy for cervical cancer: individual patient data meta-analysis | Cochrane Database Syst Rev | 2010 | 128 | 11.64(45) |
| 81 | National Cancer Data Base Analysis of Radiation Therapy Consolidation Modality for Cervical Cancer: The Impact of New Technological Advancements | Int J Radiat Oncol Biol Phys | 2014 | 127 | 18.14(26) |
| 82 | Elevated cyclooxygenase-2 expression correlates with diminished survival in carcinoma of the cervix treated with radiotherapy | Int J Radiat Oncol Biol Phys | 2001 | 127 | 6.35(82) |
| 83 | Dose effect relationship for late side effects of the rectum and urinary bladder in magnetic resonance image-guided adaptive cervix cancer brachytherapy | Int J Radiat Oncol Biol Phys | 2012 | 126 | 14(36) |
| 84 | Bladder and rectum dose defined from MRI based treatment planning for cervix cancer brachytherapy: Comparison of dose-volume histograms for organ contours and organ wall, comparison with ICRU rectum and bladder reference point | Radiother Oncol | 2003 | 126 | 7(78) |
| 85 | Vascular endothelial growth factor (VEGF) expression is a prognostic factor for radiotherapy outcome in advanced carcinoma of the cervix | Br J Cancer | 2000 | 126 | 6(86) |
| 86 | Surgery after concurrent chemoradiotherapy and brachytherapy for the treatment of advanced cervical cancer: Morbidity and outcome: Results of a multicenter study of the GCCLCC (Groupe des Chirurgiens de Centre de Lutte Contre le Cancer) | Gynecol Oncol | 2006 | 125 | 8.33(69) |
| 87 | Dose-volume histogram parameters and late side effects in magnetic resonance image-guided adaptive cervical cancer brachytherapy | Int J Radiat Oncol Biol Phys | 2011 | 124 | 12.4(44) |
| 88 | Hyperthermia dose-effect relationship in 420 patients with cervical cancer treated with combined radiotherapy and hyperthermia | Eur J Cancer | 2009 | 122 | 10.17(55) |
| 89 | Prediction of radiotherapy outcome using dynamic contrast enhanced MRI of carcinoma of the cervix | Int J Radiat Oncol Biol Phys | 2002 | 121 | 6.37(81) |
| 90 | Vaginal stenosis and sexual function following intracavitary radiation for the treatment of cervical and endometrial carcinoma | Int J Radiat Oncol Biol Phys | 1993 | 121 | 4.32(98) |
| 91 | Expression of cIAP1, a target for 11q22 amplification, correlates with resistance of cervical cancers to radiotherapy | Cancer Res | 2002 | 120 | 6.32(83) |
| 92 | Comparison between CT-based volumetric calculations and ICRU reference-point estimates of radiation doses delivered to bladder and rectum during intracavitary radiotherapy for cervical cancer | Int J Radiat Oncol Biol Phys | 2005 | 118 | 7.38(75) |
| 93 | Correlation of smoking history and other patient characteristics with major complications of pelvic radiation therapy for cervical cancer | J Clin Oncol | 2002 | 118 | 6.21(84) |
| 94 | Dynamic contrast-enhanced MR imaging of uterine cervical cancer: Pharmacokinetic analysis with histopathologic correlation and its importance in predicting the outcome of radiation therapy | Radiology | 2000 | 118 | 5.62(87) |
| 95 | Impact of dose in outcome of irradiation alone in carcinoma of the uterine cervix - analysis of 2 different methods | Int J Radiat Oncol Biol Phys | 1991 | 118 | 3.93(100) |
| 96 | Clinical outcomes of definitive intensity-modulated radiation therapy with fluorodeoxyglucose-positron emission tomography simulation in patients with locally advanced cervical cancer | Int J Radiat Oncol Biol Phys | 2010 | 117 | 10.64(52) |
| 97 | Dosimetric comparison of bone marrow-sparing intensity-modulated radiotherapy versus conventional techniques for treatment of cervical cancer | Int J Radiat Oncol Biol Phys | 2008 | 117 | 9(64) |
| 98 | Physics contributions and clinical outcome with 3d-mri-based pulsed-dose-rate intracavitary brachytherapy in cervical cancer patients | Int J Radiat Oncol Biol Phys | 2009 | 116 | 9.67(60) |
| 99 | Surgical versus radiographic determination of para-aortic lymph node metastases before chemoradiation for locally advanced cervical carcinoma - A Gynecologic oncology group study | Cancer | 2008 | 116 | 8.92(65) |
| 100 | The correlation of acute toxicity and late rectal injury in radiotherapy for cervical carcinoma: Evidence suggestive of consequential late effect (CQLE) | Int J Radiat Oncol Biol Phys | 1998 | 116 | 5.04(93) |
